# Application and Outcomes of Minimal-Dose Versus Standard-Dose Radiation in Peripheral Endovascular Intervention (KAR Endovascular Study)

**DOI:** 10.3390/jcdd12080284

**Published:** 2025-07-25

**Authors:** Subrata Kar, Clifton Espinoza

**Affiliations:** Division of Cardiovascular Medicine, Texas Tech University Health Sciences Center, Paul L. Foster School of Medicine, El Paso, TX 79905, USA; clifton.espinoza@gmail.com

**Keywords:** angioplasty, acute limb ischemia, critical limb ischemia, deep venous thrombosis, endovascular procedure, lower extremity, peripheral arterial disease

## Abstract

Background: Peripheral endovascular intervention (PEVI) is routinely performed using standard-dose radiation (SDR), which is associated with elevated levels of radiation. No study has evaluated the outcomes of minimal-dose radiation (MDR) in PEVI. Methods: We performed a prospective observational study of 184 patients (65 ± 12 years) at an academic medical center from January 2019 to March 2020 (mean follow-up of 3.9 ± 3.6 months) and compared the outcomes of MDR (n = 24, 13.0%) and SDR (n = 160, 87.0%) in PEVI. Primary endpoints included air kerma, dose area product (DAP), fluoroscopy time, and contrast use. Secondary endpoints included all-cause mortality, cardiac mortality, acute myocardial infarction, acute kidney injury, stroke, repeat revascularization, vessel dissection/perforation, major adverse limb event, access site complications, and composite of complications. Results: For MDR (68 ± 10 years, mean follow-up of 4.3 ± 5.2 months), the primary endpoints were significantly less than SDR (65 ± 12 years, mean follow-up of 3.8 ± 3.2 months; *p* < 0.001). Regarding the secondary endpoints, one vessel dissection occurred using MDR, while 36 total complications occurred with SDR (*p* = 0.037). Conclusions: PEVI using MDR was safe and efficacious. MDR showed a significant decrement in radiation parameters and fluoroscopy time. Therefore, MDR can serve as an effective alternative for PEVI in acute or critical limb ischemia.

## 1. Introduction

Peripheral endovascular intervention (PEVI) is typically performed using standard-dose radiation (SDR, fluoroscopy of ≥7.5 frames/second [f/s] and ≥7.5 f/s cine angiography) and digital subtraction angiography. Such methods are associated with high levels of radiation, which is harmful to patients and operators. It has many detrimental health consequences, such as skin injury (erythema and/or skin necrosis), left-sided brain tumors, cataracts, genetic damage, and cancer [1,2,3,4,5,6,7,8,9,10,11]. Techniques and methods to minimize radiation are paramount for operators who are exposed to lifelong radiation and patients who may have staged or repeat PEVI. Low-dose radiation utilization for cardiac catheterization and percutaneous coronary intervention (PCI) was studied in the KAR RAD study [12]. It showed a significant reduction in radiation parameters without an increase in contrast utilization. Thus, we sought to evaluate the feasibility, safety, and outcomes of patients undergoing PEVI for peripheral vascular disease (PVD) using minimal-dose radiation (MDR) compared with SDR.

## 2. Material and Methods

**Figure 1:**
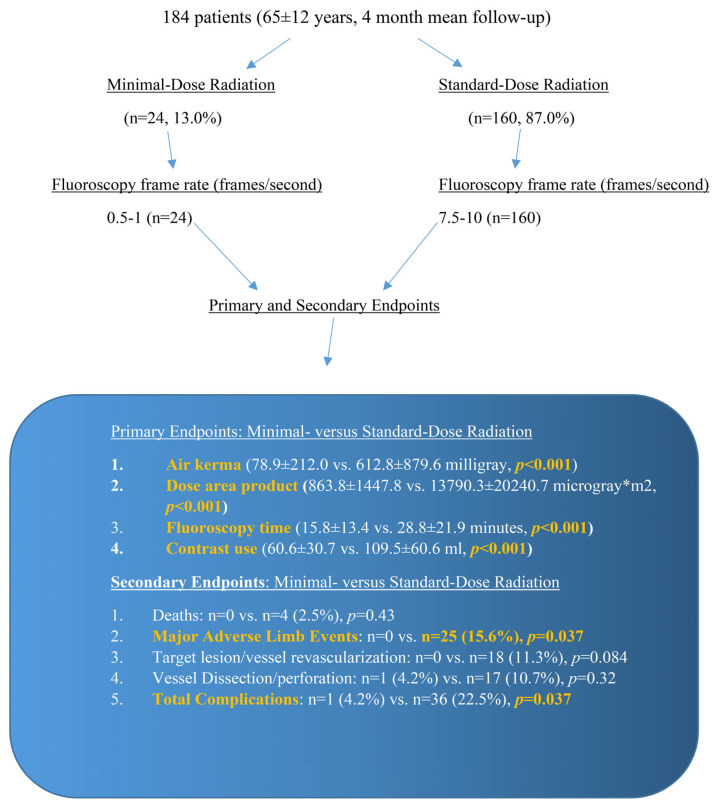
Minimal-dose radiation and standard-dose radiation protocols, with the primary and secondary outcomes specified. The primary endpoints showed a significant reduction in air kerma, dose area product, fluoroscopy time, and contrast use with the utilization of minimal-dose radiation. The secondary endpoints showed a significant reduction in major adverse limb events and total complications using minimal-dose radiation.

We conducted a prospective observational study of 184 patients between January 2019 to March 2020 at an academic university medical center and compared the outcomes of PEVI using SDR (n = 160, 87.0%, mean follow-up of 3.8 ± 3.2 months) consisting of 7.5–10 f/s fluoroscopy and 7.5–10 f/s cine angiography vs. MDR (n = 24, 13.0%, mean follow-up of 4.3 ± 5.2 months) consisting of 0.5–1.0 f/s fluoroscopy and 7.5 f/s cine angiography. Digital subtraction angiography was implemented by four experienced interventional cardiologists who also reviewed the angiographic images post-procedure. Figure 1 displays the study protocol.

One operator performed MDR PEVI without the use of SDR. The sole interventional cardiologist had previous experience using low-dose radiation and MDR in cardiac catheterization and PCI, as described in the KAR RAD study [12]. The MDR operator routinely collimated all of his images and minimized fluoroscopy time as feasible. The MDR operator also utilized ultrasound access for all of his cases. Ultrasound guidance for access did not utilize radiation, so it did not impact the radiation parameters. The remaining three operators all used SDR. This prospective study was an all-comers study, so all patients were included by the MDR operator and the SDR operators. Consequently, there were no exclusion criteria, which allows more generalizability of the results. All procedures (MDR and SDR) were performed using Siemens Artis CC x-ray equipment (Siemens Medical Solutions, Malvern, PA, USA) built in September 2010. Peripheral procedures performed in a newly constructed catheterization laboratory (2019) by the MDR operator were excluded, since the radiation levels in that lab were significantly less than the two labs built in 2010. Thus, data for the MDR operator from the new laboratory was not incorporated, since it was not comparable in radiation to the two labs built in 2010, which were used by all the operators. The baseline patient characteristics are listed in Table 1.

The primary endpoints included air kerma, dose area product (DAP), fluoroscopy time, and contrast use. Secondary endpoints included all-cause mortality, cardiac mortality, acute myocardial infarction (AMI), stroke, acute kidney injury, repeat target lesion/vessel revascularization, major adverse limb event (MALE), composite of complications, and access site complications such as major bleeding, arteriovenous fistula, pseudoaneurysm, vessel dissection/perforation, retroperitoneal bleeding, compartment syndrome, and limb amputation. MALE is defined as adverse outcomes associated with PEVI such as acute limb ischemia (ALI), limb amputation, progression from intermittent claudication to chronic limb-threatening ischemia/critical limb ischemia (CLI) or relapse to CLI, and repeat PEVI for limb ischemia [13]. Arterial dissection is an abrupt tear in the arterial wall causing an intramural accumulation of blood. This can induce tissue ischemia and/or infarction from diminished or obstructive blood flow [14]. Coronary perforation is vessel rupture, which is classified into two broad groups [15]: extravasation at the site of the intervention (Ellis type III) or perforation in a smaller distal vessel (Ellis type I and II) [15]. Vessel rupture causing perforation also applies to the peripheral vessels. AKI is defined by the Acute Dialysis Quality Initiative group as an increase in the serum creatinine of ≥50% from the baseline value and/or decrease in the glomerular filtration rate by ≥25%. It also includes a decrease in the urine output below 0.5 cc/kg/h for ≥6 h [16].

No patients were excluded from MDR by the sole operator. The MDR operator did not switch to SDR during the course of any procedure. Patient follow-up was performed via clinic evaluation. The Texas Tech University institutional review board approved our clinical research study.

### Statistical Methods

Quantitative variables were summarized using mean and standard deviation. Categorical variables were summarized using frequency and percentages. Student’s *t*-test and Chi-squared test were used for normal distributions, and Wilcoxon sum rank test was used for non-normal distributions. Non-normal distributions were converted using a natural log transformation with *p*-values representing analysis of the log-transformed variables. Dosage variables were categorized into minimal-dose intervention, standard-dose intervention, minimal-dose diagnostic, and standard-dose diagnostic. Comparison between groups were analyzed using Chi-squared and Fisher’s exact test. The main outcome variables (fluoroscopy time, DAP, air kerma, and contrast) were analyzed using a linear regression model and compared across the radiation groups while correcting for age, gender, body mass index, and diabetes. The model also compensated for clustering effects caused by patients who underwent staged or repeat intervention. Data was considered significant at a *p*-value less than 5%. The statistical validity for all the cases, both MDR and SDR, was extensively evaluated by experienced statisticians at the university, and they were validated to be accurate. The statistical analyses were performed by experienced statisticians at the university using Stata version 15 (Stata Corp LLC, Tulsa, OK, USA).

## 3. Results

MDR (n = 24, 13.0%) showed a significant reduction in the primary endpoints compared with SDR (n = 160, 87.0%). MDR air kerma (78.9 ± 212.0 milligray [mGy]) and DAP (863.8 ± 1447.8 µGy×m^2^) were significantly lower compared with SDR air kerma (612.8 ± 879.6 mGy, *p* < 0.001) and DAP (13,790.3 ± 20,240.7 uGy×m^2^, *p* < 0.001). Fluoroscopy time with MDR (15.8 ± 13.4 min) was significantly lower vs. SDR (28.8 ± 21.9 min, *p* < 0.001). Contrast use with MDR was also significantly lower compared with SDR (60.6 ± 30.7 vs. 109.5 ± 60.6, *p* < 0.001). The MDR operator was experienced in using MDR based on the KAR RAD study, so he did not use frequent contrast injections for fluoroscopy or cineangiography images, which reduced his contrast utilization, irrespective of case complexity. For MDR, Transatlantic Intersociety Consensus (TASC) Classification D and Rutherford Grade IV procedures were significantly more prevalent compared with SDR (20.8% vs. 5.0% and 4.2% vs. 0%, respectively), which denotes the most complex and symptomatic cases. Nevertheless, the MDR operator was able to perform all the procedures regardless of case complexity without an increase in contrast. His past experience from the KAR RAD study was useful. Table 2 shows the primary endpoints of MDR and SDR in diagnostic and interventional procedures.

During peripheral diagnostic angiography using MDR (n = 7, 29.2%) vs. SDR (n = 32, 20%), air kerma (67.0 ± 147.7 vs. 550.8 ± 431.2 mGy; *p* < 0.001) and DAP (1233.5 ± 2494.6 vs. 12,432.3 ± 8759.1 uGy×m^2^; *p* < 0.001) were significantly reduced. Fluoroscopy time (7.9 ± 8.9 vs. 16.2 ± 18.9 min; *p* = 0.007) and contrast use (42.6 ± 28.0 vs. 81.5 ± 44.9; *p* = 0.013) was also significantly diminished using MDR vs. SDR. During PEVI in MDR (n = 17, 70.8%) vs. SDR (n = 128, 80.0%), air kerma (83.8 ± 237.3 vs. 627.3 ± 955.5 mGy; *p* < 0.001) and DAP (711.6 ± 771.5 vs. 14,108.6 ± 22,099.9 uGy×m^2^; *p* < 0.001) were also significantly diminished. Fluoroscopy time (19.0 ± 13.8 vs. 31.8 ± 21.5 min; *p* < 0.001) and contrast use (68.1 ± 29.4 vs. 116.3 ± 62.2; *p* = 0.006) were also significantly decreased using MDR vs. SDR (Table 2).

The secondary endpoints (Table 3) showed that MDR was safe and did not increase complications. The composite of complications using MDR (n = 1, 4.2%) was significantly less than SDR (n = 36, 22.5%; *p* = 0.037). With MDR, 1 arterial dissection occurred (n = 1, 4.2%) while 17 arterial dissections/perforations occurred with SDR (10.6%; *p* = 0.32). Target lesion/vessel revascularization occurred in 0 patients undergoing MDR, whereas it occurred in 18 undergoing SDR (11.3%, *p* = 0.084). MALE occurred in none of the MDR patients, while it occurred in 25 of the SDR group (13.9%, *p* = 0.037). More patients who underwent SDR had acute renal failure post-PEVI (n = 24, 15.0%) compared with MDR (n = 0, *p* = 0.042).

**Figure 2:**
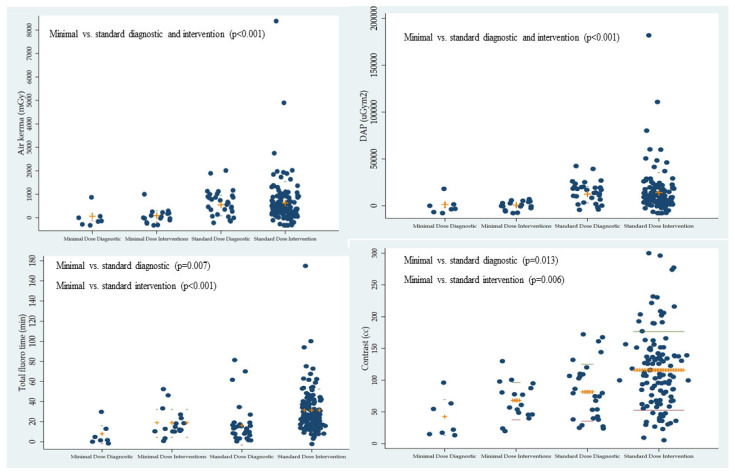
Comparison of the radiation parameters (air kerma, dose area product, and total fluoroscopy time) and contrast use with minimal- or standard-dose radiation during peripheral diagnostic and endovascular intervention.

No deaths occurred using MDR, while four deaths occurred using SDR (2.5%; *p* = 0.43). One patient presented with ALI with a non-viable limb after revascularization complicated by contrast-induced nephropathy requiring hemodialysis. A second patient expired from sepsis post-carotid artery stenting with a non-ST-segment myocardial infarction. A third patient developed iliac vein thrombosis and perforation 21 days post-venous stent implantation for deep venous thrombosis with post-thrombotic syndrome. The patient expired from erosion of the venous stent inducing iliac vein perforation. The last patient expired 7 days after PEVI for CLI complicated by post-PEVI myocardial infarction (MI) and cardiac arrest. The patient expired in the catheterization laboratory during PCI. Figure 2 shows the comparison of radiation parameters and contrast use in MDR or SDR during diagnostic and interventional procedures.

The most common indications for PEVI were chronic limb-threatening ischemia/CLI (n = 89, 48.4%), ALI (n = 30, 16.3%), and intermittent claudication (n = 27, 14.7%). In the MDR group, 17 patients underwent PEVI (70.8%). All of these patients had symptomatic peripheral arterial disease (PAD), lower extremity gangrene/ulcerations, or symptomatic peripheral venous disease (PVD). In the SDR group, 128 patients underwent PEVI (80.0%). In the MDR group, ALI and CLI occurred in 18 patients (75.0%) vs. 63.1% (n = 101) in the SDR group. No significant difference was noted in the number patients undergoing MDR vs. SDR PEVI for ALI (*p* = 0.85) or CLI (*p* = 0.057). Most of the patients underwent infrainguinal (n = 92, 50.0%) and infrapopliteal PEVI (n = 66, 35.9%). No significant difference was noted in the number of patients undergoing suprainguinal, infrainguinal, or infrapopliteal PEVI using MDR or SDR. Chronic total occlusion (CTO) intervention was performed in 29.3% (n = 54), of which 37.5% were performed using MDR (n = 9) and 28.1% using SDR (n = 45; *p* = 0.35). Balloon angioplasty was performed for infrainguinal and infrapopliteal PAD. Drug-coated balloon angioplasty (infrainguinal) was performed at the discretion of the operator due to a meta-analysis from 2018 reporting increased risk of mortality with the use of paclitaxel drug-coated balloons or stents in the femoropopliteal arteries [17]. The Food and Drug Administration also reported the aforementioned results in 2019. In suprainguinal PAD, balloon angioplasty followed by stent implantation was performed. In venous intervention, balloon angioplasty followed by stent implantation was routinely performed using intravascular ultrasound. Retrograde femoral access was the most common approach (n = 177, 96.2%). The TASC, Rutherford, and Fontaine classifications for PAD are listed in Table 1.

The most common risk factors included the following: hypertension (n = 138, 75.0%), diabetes (n = 124, 67.4%), hyperlipidemia (n = 98, 53.3%), current or past history of smoking (n = 90, 48.9%), history of coronary artery disease (n = 57, 31.0%), and/or history of MI/PCI (n = 49, 26.6%).

## 4. Discussion

PEVI is associated with an excess amount of radiation, which is detrimental to patients and operators [1,4,5,7,11]. PVD is associated with possible repeat intervention due to the underlying disease and patient comorbidities such as obesity, smoking, diabetes, hyperlipidemia, chronic kidney disease, and hypertension. Radiation dose is disproportionately high in PEVI, with the potential for tissue injury occurring in 1 out of 14 patients and an associated risk of malignancy [18]. PEVI performed in the pelvis has been associated with higher radiation exposure, which can be comparable to computed tomography of the abdomen and pelvis [9,19]. Therefore, methods to reduce radiation are vital for the lifelong exposure of patients and also for physicians who consistently perform these procedures. A retrospective study of 87 patients undergoing peripheral vascular intervention showed that 7.5 f/s fluoroscopy reduced DAP compared with 15 f/s (air kerma was not assessed) [20]. However, no study has evaluated the outcomes of patients undergoing PEVI using MDR, nor compared such patients with SDR.

Our study showed that MDR was feasible, efficacious, and safe for patients with PAD, including ALI, CLI, and CTO, along with PVD. MDR PEVI was used in suprainguinal, infrainguinal, and infrapopliteal PAD with no significant difference in the number of patients undergoing SDR PEVI for those lesions. No patients were excluded for MDR PEVI. Complications were not significantly increased compared with SDR, nor was fluoroscopy time or contrast use. Thus, MDR was an effective method to significantly reduce radiation parameters including air kerma, DAP, and fluoroscopy time, which is favorable for index and subsequent procedures. This is particularly valuable for patients who may inevitably undergo repeat, staged, or CTO interventional procedures. Furthermore, MDR PEVI is also beneficial for the operator, since it will reduce their lifetime radiation dose, which will diminish the stochastic effects of radiation.

MDR can significantly reduce radiation to the patient, which reduces scatter radiation to the operator. This is significantly beneficial from a health perspective, reducing the harmful effects of radiation to the entire cardiac catheterization staff. The immediate impact of using MDR for procedures is decreasing the deterministic effects of radiation, such as skin injury causing erythema and/or skin necrosis. Long-term use of MDR can mitigate the stochastic effects of radiation, such as cancer for patients and operators. Image quality was preserved using MDR by applying collimation, magnification, and wedge filters.

Radiation absorption during fluoroscopy is detrimental to patients and operators such as interventional cardiologists, electrophysiologists, general cardiologists, interventional radiologists, and vascular surgeons. Techniques to minimize radiation are critical for patients and invaluable for operators, who are exposed to lifelong radiation. Our study showed that radiation reduction can be accomplished using MDR without compromising patient safety compared with SDR. Therefore, utilizing MDR for PEVI can have a dramatic impact on patient/operator radiation exposure, diminish the stochastic and deterministic effects of radiation, and possibly enhance the longevity of the operator’s career by mitigating adverse health consequences from radiation.

Patients are exposed to a lifetime accumulation of radiation from cardiac, peripheral, and various interventional procedures, along with diagnostic cardiac or radiology imaging tests, which increase their radiation exposure. Hence, attempts to reduce radiation are vital to patient health. Our prospective study is an important addition to the literature, because it highlights the importance of radiation reduction. It also showed that radiation reduction can be performed in a safe manner without additional resources or equipment, which can be a limitation due to monetary constraints. Constructing new catheterization laboratories or purchasing radiation reduction equipment is an expensive and time-consuming endeavor, which is not feasible in some institutions.

### Limitations

The study was performed in a single tertiary care center. One interventional cardiologist performed MDR PEVI, while multiple interventional cardiologists performed SDR PEVI, so the numbers of cases were unequal. This is a limitation of our study. Nevertheless, this is a meaningful study, since all the patients were included for MDR PEVI (all-comers study), so it provides practical real-world data on the feasibility and benefits of MDR PEVI. Furthermore, none of the patients who underwent MDR PEVI was switched to the SDR group, even for complex PEVI procedures including CTO interventions; hence, the applicability and potential feasibility of MDR with previous experience using MDR, as reported in the KAR RAD study [12]. The MDR operator’s past experience using low-dose and MDR in cardiac catheterization and PCI (KAR RAD Study) [12] was advantageous for PEVI.

## 5. Conclusions

To our knowledge, this is the first prospective study utilizing MDR for PEVI in PVD and ALI or chronic limb-threatening ischemia/CLI. MDR showed a significant reduction in radiation endpoints (fluoroscopy time, air kerma, and DAP). MDR was not associated with increased complications or contrast use, so it was safe for PAD and venous disease. It is feasible for urgent and emergent procedures such as ALI or CLI. Consequently, MDR can be a valuable tool to curtail radiation, which is beneficial for patients, operators, and the entire healthcare staff in the catheterization laboratory. Further clinical research using MDR with long-term data is necessary.

## Figures and Tables

**Table 1:** Baseline patient characteristics for minimal- and standard-dose radiation.

	Total	Minimal	Standard	*p*-Value
n (%)	184	24 (13.0%)	160 (87.0%)	
Age, mean (SD*)	65.0 (11.9)	67.8 (9.6)	64.6 (12.2)	0.22
Weight (kg), mean (SD*)	76.4 (17.1)	74.7 (14.7)	76.7 (17.4)	0.59
Height (cm), mean (SD*)	163.9 (12.4)	163.4 (7.0)	163.9 (13.0)	0.84
Body Mass Index (kg/m^2^), mean (SD*)	28.3 (6.0)	28.0 (5.0)	28.3 (6.2)	0.79
Body Surface Area (m^2^), mean (SD*)	3.7 (17.6)	1.8 (0.2)	4.0 (18.9)	0.59
Fluoroscopy Time (min), mean (SD*)	27.1 (21.4)	15.8 (13.4)	28.8 (21.9)	<0.001
DAP† (uGy×m^2^), mean (SD*)	12,085.7 (19,361.4)	863.8 (1447.8)	13,790.3 (20,240.7)	<0.001
Air Kerma (mGy), mean (SD*)	542.4 (842.4)	78.9 (212.0)	612.8 (879.6)	<0.001
Contrast (mL), mean (SD*)	102.2 (59.7)	60.6 (30.7)	109.5 (60.6)	<0.001
Follow-up (months), mean (SD*)	3.9 (3.6)	4.3 (5.2)	3.8 (3.2)	0.52
Risk Factors Diabetes	124 (67.4%)	20 (83.3%)	104 (65.0%)	0.074
Hypertension	138 (75.0%)	20 (83.3%)	118 (73.8%)	0.31
Hyperlipidemia	98 (53.3%)	11 (45.8%)	87 (54.4%)	0.43
Past Medical History of CAD‡	57 (31.0%)	10 (41.7%)	47 (29.4%)	0.22
Past History of MI/PCI§	49 (26.6%)	9 (37.5%)	40 (25.0%)	0.20
Atrial fibrillation/Atrial Flutter	13 (7.1%)	5 (20.8%)	8 (5.0%)	0.005
History of CKD/ESRD||, On Hemodialysis	6 (3.3%)	1 (4.2%)	5 (3.1%)	0.79
History of Peripheral Intervention	46 (25.0%)	9 (37.5%)	37 (23.1%)	0.13
History of Vascular Surgery	5 (2.7%)	2 (8.3%)	3 (1.9%)	0.070
History of Acute/Critical Limb Ischemia	29 (15.8%)	7 (29.2%)	22 (13.8%)	0.053
Current/Former Smoker	90 (48.9%)	9 (37.5%)	81 (50.6%)	0.23
History of Amputation	28 (15.2%)	6 (25.0%)	22 (13.8%)	0.15
Procedural Indications				
Intermittent Claudication	27 (14.7%)	5 (20.8%)	22 (13.8%)	0.36
Critical Limb Ischemia	89 (48.4%)	15 (62.5%)	74 (46.3%)	0.14
Acute Limb Ischemia	30 (16.3%)	3 (12.5%)	27 (16.9%)	0.59
Lower Extremity Edema/DVT{/May Thurner/CTO# Vein	25 (13.6%)	1 (4.2%)	24 (15.0%)	0.15
Venous Stenosis	2 (1.1%)	0 (0.0%)	2 (1.3%)	0.58
Left Heart Catheterization with Peripheral Angiogram	6 (3.3%)	0 (0.0%)	6 (3.8%)	0.33
Peripheral Aneurysm	4 (2.2%)	0 (0.0%)	4 (2.5%)	0.43
Interventional Location and Access Site Suprainguinal Intervention	40 (21.7%)	2 (8.3%)	38 (23.8%)	0.088
Infrainguinal Intervention	92 (50.0%)	8 (33.3%)	84 (52.5%)	0.080
Infrapopliteal Intervention	66 (35.9%)	10 (41.7%)	56 (35.0%)	0.53
Femoral Access	156 (84.8%)	24 (100.0%)	132 (82.5%)	0.026
Pedal Access	18 (9.8%)	0 (0.0%)	18 (11.3%)	0.084
Brachial/Radial Access	28 (15.2%)	0 (0.0%)	28 (17.5%)	0.026
Retrograde Approach	177 (96.2%)	23 (95.8%)	154 (96.3%)	0.92
Antegrade Approach	1 (0.5%)	1 (4.2%)	0 (0.0%)	0.010
Peripheral Intervention	144 (78.3%)	17 (70.8%)	127 (79.4%)	0.34
CTO# Intervention	54 (29.3%)	9 (37.5%)	45 (28.1%)	0.35
Venous Intervention	19 (10.3%)	1 (4.2%)	18 (11.3%)	0.29
Staged Intervention	17 (9.2%)	3 (12.5%)	14 (8.8%)	0.55
TASC** Classification A	16 (8.7%)	4 (16.7%)	12 (7.5%)	0.14
TASC** Classification B	54 (29.3%)	9 (37.5%)	45 (28.1%)	0.35
TASC** Classification C	56 (30.4%)	3 (12.5%)	53 (33.1%)	0.041
TASC** Classification D	13 (7.1%)	5 (20.8%)	8 (5.0%)	0.005
Rutherford Grade I	27 (14.7%)	5 (20.8%)	22 (13.8%)	0.36
Rutherford Grade II	55 (29.9%)	10 (41.7%)	45 (28.1%)	0.18
Rutherford Grade III	51 (27.7%)	5 (20.8%)	46 (28.7%)	0.42
Ruth Grade IV	1 (0.5%)	1 (4.2%)	0 (0.0%)	0.01
Fontaine Stage I	0%			
Fontaine Stage II	33 (17.9%)	5 (20.8%)	28 (17.5%)	0.69
Fontaine Stage III	37 (20.1%)	7 (29.2%)	30 (18.8%)	0.24
Fontaine Stage IV	50 (27.2%)	6 (25.0%)	44 (27.5%)	0.8

SD* = Standard Deviation; DAP† = Dose Area Product; CAD‡ = Coronary Artery Disease; MI/PCI§ = Myocardial Infarction/Percutaneous Coronary Intervention; CKD/ESRD|| = Chronic Kidney Disease/End Stage Renal Disease; DVT{ = Deep Venous Thrombosis; CTO# = Chronic Total Occlusion; TASC** = Transatlantic Intersociety Consensus.

**Table 2:** Primary endpoints for diagnostic and peripheral endovascular intervention.

Peripheral Diagnostic Angiogram	Minimal Dose	Standard Dose	*p*-Value
n (%)	7 (29.2%)	32 (20.0%)	
Age, mean (SD*)	64.4 (6.5)	64.7 (9.4)	0.95
Weight (kg), mean (SD*)	74.4 (16.6)	74.9 (18.1)	0.95
Height (cm), mean (SD*)	164.7 (6.2)	162.5 (10.6)	0.59
Body Mass Index (kg/m^2^), mean (SD*)	27.3 (6.2)	28.3 (6.4)	0.71
Body Surface Area (m^2^), mean (SD*)	1.8 (0.2)	7.1 (29.9)	0.65
Fluoroscopy Time (min), mean (SD*)	7.9 (8.9)	16.2 (18.9)	0.007
DAP† (uGy×m^2^), mean (SD*)	1233.5 (2494.6)	12,432.3 (8759.1)	<0.001
Air Kerma (mGy), mean (SD*)	67.0 (147.7)	550.8 (431.2)	<0.001
Contrast (mL), mean (SD*)	42.6 (28.0)	81.5 (44.9)	0.013
Follow-up (months), mean (SD*)	3.8 (6.3)	3.3 (2.8)	0.43
**Peripheral Intervention**	**Minimal Dose**	**Standard Dose**	***p*-Value**
n (%)	17 (70.8%)	128 (80.0%)	
Age, mean (SD*)	69.1 (10.5)	64.5 (12.8)	0.16
Weight (kg), mean (SD*)	74.8 (14.5)	77.1 (17.3)	0.59
Height (cm), mean (SD*)	162.8 (7.5)	164.3 (13.6)	0.67
Body Mass Index (kg/m^2^), mean (SD*)	28.3 (4.5)	28.3 (6.2)	0.96
Body Surface Area (m^2^), mean (SD*)	1.8 (0.2)	3.2 (15.0)	0.72
Fluoroscopy Time (min), mean (SD*)	19.0 (13.8)	31.8 (21.5)	<0.001
DAP† (uGy×m^2^), mean (SD*)	711.6 (771.5)	14,108.6 (22,099.9)	<0.001
Air Kerma (mGy), mean (SD*)	83.8 (237.3)	627.3 (955.5)	<0.001
Contrast (mL), mean (SD*)	68.1 (29.4)	116.3 (62.2)	0.006
Follow-up (months), mean (SD*)	4.5 (4.9)	3.9 (3.3)	0.90

SD* = Standard Deviation; DAP† = Dose Area Product.

**Table 3:** Secondary endpoints in the study population.

	Total	Minimal	Standard	*p*-Value
**Variables (n, %)**	184	24 (13.0%)	160 (87.0%)	
Total Complications	37 (20.1%)	1 (4.2%)	36 (22.5%)	0.037
Major Adverse Limb Event	25 (13.6%)	0 (0.0%)	25 (15.6%)	0.037
Myocardial Infarction	4 (2.2%)	0 (0.0%)	4 (2.5%)	0.43
Stroke	1 (0.5%)	0 (0.0%)	1 (0.6%)	0.70
Dissection/Perforation	18 (9.8%)	1 (4.2%)	17 (10.6%)	0.32
Acute Kidney Injury	24 (13.0%)	0 (0.0%)	24 (15.0%)	0.042
Target Lesion/vessel Revascularization	18 (9.8%)	0 (0.0%)	18 (11.3%)	0.084
Death	4 (2.2%)	0 (0.0%)	4 (2.5%)	0.43
**Peripheral Diagnostic Angiogram**	**Minimal Dose**	**Standard Dose**		
Total Complications	0 (0%)	2 (6%)		0.50
Major Adverse Limb Event	0%	0%		
Myocardial Infarction	0 (0%)	1 (3%)		0.64
Stroke	0 (0%)	1 (3%)		0.64
Dissection/Perforation	0%	0%		
Acute Kidney Injury	0 (0%)	3 (9%)		0.40
Target Lesion/Vessel Revascularization	0%	1 (3%)		0.64
Death	0%	1 (3%)		0.64
**Peripheral Endovascular Intervention**	**Minimal Dose**	**Standard Dose**		
Total Complications	1 (5.9%)	34 (26.6%)		0.061
Major Adverse Limb Event	0 (0.0%)	25 (19.5%)		0.045
Myocardial Infarction	0 (0.0%)	3 (2.3%)		0.52
Stroke	0%	0%		
Dissection/Perforation	1 (5.9%)	17 (13.3%)		0.38
Acute Kidney Injury	0 (0.0%)	21 (16.4%)		0.071
Target Lesion/vessel Revascularization	0 (0.0%)	17 (13.3%)		0.11
Death	0 (0.0%)	3 (2.3%)		0.52

## Data Availability

The original contributions presented in this study are included in the article. Further inquiries can be directed to the corresponding author.
